# Comprehensive Time-Course Transcriptome and Co-expression Network Analyses Identify Salt Stress Responding Mechanisms in *Chlamydomonas reinhardtii* Strain GY-D55

**DOI:** 10.3389/fpls.2022.828321

**Published:** 2022-02-24

**Authors:** Luo-Yan Zhang, Zhao-Tian Xing, Li-Qian Chen, Xue-Jie Zhang, Shou-Jin Fan

**Affiliations:** Shandong Provincial Key Laboratory of Plant Stress Research, College of Life Sciences, Shandong Normal University, Jinan, China

**Keywords:** *Chlamydomonas reinhardtii*, time-course transcriptome, salt stress, co-expression network, strain GY-D55

## Abstract

It is highly necessary to understand the molecular mechanism underlying the salt stress response in green algae, which may contribute to finding the evolutionary cues of abiotic stress response in plants. Here, we reported a comprehensive temporal investigation of transcriptomes using data at eight different time points, from an early stage (2 h) to a late stage (up to 96 h) in *Chlamydomonas reinhardtii* GY-D55 cells. The principal component analysis (PCA) of transcriptome profiles showed that the samples of the early and late stages were well separated. A total of 12,445 genes were detected as differentially expressed genes. There were 1,861/2,270 common upregulated/downregulated genes for each time point compared with control samples. Samples treated with salt for 2, 8, and 24 h had a relatively large number of characteristic upregulated/downregulated genes. The functional enrichment analysis highlighted the timing of candidate regulatory mechanisms for salt stress responses in GY-D55 cells. Short time exposure to salt stress impaired oxidation-reduction, protein synthesis and modification, and photosynthesis. The algal cells promoted transcriptional regulation and protein folding to deal with protein synthesis/modification impairments and rapidly accumulated glycerol in the early stage (2–4 h) to cope with osmotic stress. At 12 and 24 h, GY-D55 cells showed increased expressions of signaling and photosynthetic genes to deal with the damage of photosynthesis. The co-expression module blue was predicted to regulate endoplasmic reticulum (ER) stress at early time points. In addition, we identified a total of 113 transcription factors (TFs) and predicted the potential roles of Alfin, C2C2, and the MYB family TFs in algal salt stress response.

## Introduction

As one of the most critical abiotic stresses, high salinity greatly impairs agricultural productivity globally. Although plants have progressively acquired a variety of adaptive molecular, physiological, and biochemical mechanisms to deal with salinity stress, it can still affect 30% of arable soils by 2050 (Munns and Tester, 2008). The physiological/biochemical regulation, transcription/translation machinery, and epigenetic changes may trigger ecological adaptation (such as salt stress adaptation), while these changes over time can remarkably affect species phenotypes and distributions (Yang et al., 2019; Funck et al., 2020; Verdu et al., 2021). Gene-rich mixotrophic lineages, such as *Chlamydomonas*, can quickly adapt to either climate change-induced or physical abiotic stress. Therefore, it is necessary to explore the molecular mechanism underlying the salt stress response in green algae, which may contribute to identifying the evolutionary cues of abiotic stress response in plants and developing salt-resistant crops with valuable salt-responding characteristics (Husic and Quigley, 1990; Perrineau et al., 2014; Gierz et al., 2017; Wang et al., 2018, 2020; Yang et al., 2020).

Different organisms adapt to extreme surrounding conditions *via* various mechanisms, and understanding these mechanisms can help explore the key features of biological processes (BPs), including the biochemical limits to macromolecular stability and the genetic instructions for constructing macromolecules that stabilize under one or more extreme conditions (Grant et al., 1998; Luo and Liu, 2011; Foflonker et al., 2014). Generally speaking, there are two basic mechanisms in halophiles: (i) “salt-in” strategy: halophilic algae accumulate KCl in their cells to keep high intracellular salt levels, osmotically at least equivalent to the external levels; (ii) other halophilic algae create or accumulate osmoregulation substances that have osmotic potential. The majority of green algae (Chlorophyceae) are moderately halophilic. However, a few algae, including *Dunaliella salina*, are extremely halophilic species (Grant et al., 1998; Gammoudi et al., 2021). These species are responsible for most of the primary production when exposed to high salt stress (Oren, 2005). Nevertheless, most freshwater strains show retarded development and impaired survival when exposed to high salinity, and these strains are generally unable to survive beyond a low threshold of salinity. Various pathways are used by these salt-sensitive algae to deal with salt stress. Glycolysis, the Kennedy pathway, and the Calvin cycle are all adopted to enhance both osmolyte production and reserves of storage molecules, mostly lipids (Shetty et al., 2019). A previous study on salt-adapted mutants of *Chlamydomonas reinhardtii* has shown that algae can adapt to loss in photosynthetic efficiency by taking up acetate to enhance energy generation and carbon assimilation (Perrineau et al., 2014).

As a free-living freshwater green alga, the unicellular alga *C. reinhardtii* is a well-characterized model alga, and its full genome has been sequenced. This alga is widely used to investigate stress-inducible responses since it carries a series of stress-related genes. However, the functional significance of these genes in adaptive metabolism remains largely undetermined (Mendez-Alvarez et al., 1999; Grossman, 2000; Perrineau et al., 2014). *C. reinhardtii* has been used to assess responses to a series of abiotic stress agents, including salinity, temperature, irradiation, nutrient starvation, and heavy metals (Hanikenne, 2003; Pollock et al., 2005; Mahong et al., 2012; James et al., 2013; Neelam and Subramanyam, 2013; Perrineau et al., 2014; Erickson et al., 2015; Breker et al., 2016).

Existing research on *C. reinhardtii* shows that at the morphological level, salt stress of 150 or 200 mM NaCl results in flagellar resorption, decreased cell size, slower growth rate, and palmelloid morphology (Neelam and Subramanyam, 2013; Khona et al., 2016). Salt stress metabolically affects the demand for energy to keep ion homeostasis, the scavenging of reactive oxygen species (ROS) by antioxidant enzymes, and the upregulation of heat shock proteins (HSPs) and chaperones to aid protein renaturation due to misfolding or aggregation (Yokthongwattana et al., 2012; Sithtisarn et al., 2017). Salinity composes stress by damaging ionic and osmotic balances in plants. Algal cells with inflexible cell walls have limited ability to change cell volume and thus depend heavily on organic solutes for osmoregulation. Glycerol, proline, and trehalose with a neutral charge and low toxicity at high concentrations are usually used as compatible solutes. Glycerol is an effective compatible solute that is produced by most salt-sensitive algal species under high saline stress. In *Chlorella autotrophica* (Ahmad and Hellebust, 1984), *C. reinhardtii* (Perrineau et al., 2014), *Chlamydomonas mexicana* (Salama el et al., 2013), *Chlamydomonas pulsatilla*, and *Chlamydomonas* sp. JSC4 (Ho et al., 2017) showed an increase in glycerol content in response to increasing salinity. Besides, salt stress usually induces the expression of many transcription factors (TFs), which then activate the expression of numerous downstream osmotic and ionic stress responding genes (Gong et al., 2020). In *C. reinhardtii*, basic leucine region zipper (bZIP) TFs play important roles in mediating photosynthesis and lipid accumulation of alga in response to stresses. Several members from bZIP, C3H, Dof, and MYB TFs families were differentially expressed throughout the entire time course in cold stress responding (Li et al., 2020).

Some studies have reported that different strains of this green alga have various salt stress responses. In *C. reinhardtii*, a retarded cell growth and weaker photosynthesis are observed after a short time of exposure to sodium chloride (NaCl), whereas the growth and photosynthetic activity in several strains of *C. reinhardtii* recover over 24 h (Perrineau et al., 2014). In a long-term selection study, evolved *C. reinhardtii* populations can grow rapidly in a high salt medium (200 mM NaCl) as progenitor cells (Perrineau et al., 2014). These *C. reinhardtii* cells display well-known responses to salt stress, such as reduced photosynthesis, enhanced glycerophospholipid signaling, and activated transcription and translation machinery. Quantitative proteomic comparison of salt stress in *C. reinhardtii* strain CC-4325 and the snow alga *Chlamydomonas nivalis* reveals that salt stress enhances the accumulation of fatty acids in the un-sequenced alga *C. nivalis* as it switches to a non-growth state, while *C. reinhardtii* does not have this response (Hounslow et al., 2021). In our previous study, we have predicted the molecular and genetic mechanisms of salt stress responses in *C. reinhardtii* strain GY-D55 after short-term acclimation to salt stress (200 mM NaCl for 24 h) (Wang et al., 2018).

It is highly possible that many genes that are important for the salt stress responding in the *C. reinhardtii* strain GY-D55 (but are expressed only in a short period of time) have not been identified yet, due to the fact that the previous proteomic and transcriptomic studies did not have sufficient temporal resolution. Here we report a comprehensive temporal investigation of transcriptomes using data for eight time points, from early stage (2 h) to late stage (up to 96 h). This time-course transcriptome analysis aims to highlight the timings of candidate regulatory genes and mechanisms for salt stress responses in GY-D55 cells.

## Materials and Methods

### Algal Material Preparation, Stress Treatments, and Morphological Observations

The wild-type *C. reinhardtii* strain GY-D55 was obtained from LeadingTec Co., Ltd.^1^ and maintained in 150 ml of BG-11 medium on a shaking table at 120 rpm under the following conditions: photoperiod 16-8 h/light-dark, temperature 23°C, and light intensity 100 μmol m^–2^ ^s–1^. Under the above-mentioned conditions, *C. reinhardtii* cells were cultured in a BG11 medium for approximately 14 days until the cell density reached 2 × 10^6^ cells/ml. The cells of the mid-logarithmic phase were used for salt stress treatment referring to the study published by Khona et al. (2016).

It was necessary to point out how we set up the time points of salt stress in this study. The existing studies have suggested that there are certain dynamic changes in the phenotype, physiology, and biochemistry affected in *C. reinhardtii* cells by salt stress at different time points, which helped us design time points. In the study published by Vega et al. (2006), the photosynthetic activity in *C. reinhardtii* is initially (1–2 h) blocked by the addition of 200 mM salt. During 4–12 h, the decreasing trend of photosynthetic activities slowed down and respiratory activities began to recover gradually (Vega et al., 2006). These findings suggest that there is a dynamic inhibitory effect on the photosynthesis in *C. reinhardtii* during 2–12 h. A duration of 24 h is the key time point for the alga to respond to salt stress, and the photosynthetic activity of several strains recovers over 24 h (Vega et al., 2006; Crespo, 2012; Erickson et al., 2015). In the study published by Ji et al. (2018), the total lipid content in *C. reinhardtii* cells is significantly increased when exposed to salt stress (150 mM) for 48 h. Besides, 200 mM NaCl treatment inhibits the growth and significantly increases the lipid content of *C. reinhardtii* at 3 days (72 h) (Hang et al., 2020). Salt treatment strikingly induces the production of nitric oxide (NO) after 1–3 days (24–72 h), suggesting that NO, as another second messenger, triggers salt stress response (Chen et al., 2016; Bazzani et al., 2021). Besides, the salt stress gradually induced the activities of antioxidant enzymes after 1 or 3 days (72 h) of salt treatment, and such induced activities were declined after 3 days (72–96 h) of salt treatment. Therefore, it was reasonable to set an eight-time-point regimen (2, 4, 8, 12, 24, 48, 72, and 96 h) to explore the molecular mechanisms of photosynthesis impairments and the suppression of ROS and lipid metabolism in salt stress response in *C. reinhardtii* cells.

The NaCl treatment was carried out as previously described by Wang et al. (2018). Briefly, a 50 ml medium containing 800 mM NaCl was added to a 150 ml culture medium, the final NaCl concentration was 200 mM, and the pH of the medium was adjusted to 7.0. Cells cultured in the absence of NaCl were used as the control group. Each experiment was conducted in triplicate. The cells of *C. reinhardtii* were collected at eight preset time points after exposure to 200 mM NaCl: 2, 4, 8, 12, 24, 48, 72, and 96 h. To evaluate the morphological changes, including the difference in cell size, salt stress-induced palmelloids, or chlorophyll bleaching under stressful conditions, a 50 μL sample was observed using an Olympus SZ61 microscope (Olympus Instruments Inc., Tokyo, Japan) at 40/100× magnification. The *t*-test analysis was used to evaluate the growth ratio between cells under control and saline condition. The cell count of algal material was estimated at eight time-points and relative growth rates (RGR) were calculated: RGR = (lnN2 − lnN1)/Δt × 100%, where N1 is the cell count at time-point 1 and N2 is the cell count at time-point 2, Δt is the time interval (hours). The statistical differences between salt stress and control samples were analyzed by the one-way ANOVA in R.

### RNA Extraction, Illumina Library Construction, and Sequencing

Briefly, 100 ml cell cultures from different groups were subjected to centrifugation at 3,000 *g* for 5 min, and the cell pellet was resuspended in 25 ml RNAlater solution (Ambion). The total RNA was isolated using the TRIzol Reagent (Invitrogen) according to the manufacturer’s instructions. The integrity of RNA was evaluated using the RNA Nano 6000 Assay Kit of the Agilent Bioanalyzer 2100 system (Agilent Technologies, CA, United States) and the NanoDrop 2000 spectrophotometer (Thermo Scientific, Wilmington, MA, United States).

Sequencing libraries were constructed using the NEBNext^®^ UltraTM RNA Library Prep Kit for Illumina^®^ (NEB, United States) according to the manufacturer’s instructions, and the sequences were attributed to each sample based on the index codes. Briefly, total RNA was subjected to purification using poly-T oligo attached magnetic beads. Purified messenger RNA (mRNA) was reversely transcribed into first-strand complementary DNA (cDNA) using a random hexamer primer and M MuLV Reverse Transcriptase (RNase H^–^). DNA Polymerase I and RNase H were adopted for the synthesis of the second-strand cDNA. cDNA fragments of 370–420 bp were subjected to purification using the AMPure XP system (Beckman Coulter, Beverly, MA, United States). The size selected, adaptor-ligated fragments were purified isolated and enriched by PCR. The resulting products were subjected to high throughput sequencing using an Illumina HiSeq X platform (Illumina, San Diego, CA, United States). All genetic data have been submitted to the NCBI Sequence Read Archive (SRA) database,^2^ PRJNA770825 and the Gene Expression Omnibus (GEO) database,^3^ GSE191218.

### RNA Sequencing and Read Mapping of the *Chlamydomonas reinhardtii* Transcriptome

The RNA sample of every accession was sequenced by the Illumina NovaSeq 6000. The cDNA library was constructed, and Illumina pair-end 150 bp sequencing (PE150) was carried out at Novogene Co., Ltd.^4^ Raw reads of the fastq format were firstly processed through in-house perl scripts. In this step, the adapter containing reads, ploy-N containing reads, and low-quality reads were removed from raw data, yielding clean reads. Meanwhile, the Q20, Q30, GC content, and sequence duplication levels of the clean data were determined. All the downstream analyses were conducted using high-quality clean data. Reference genome^5^ and gene model annotation files^6^ were downloaded from the genome website directly. The index of the reference genome was built using Hisat2 (v2.0.5), which was adopted to align paired-end clean reads to the reference genome. For the prediction of novel transcripts, StringTie (v1.3.3b) was adopted to assemble the mapped reads of each sample as previously described (Pertea et al., 2015).

### Calculation of Gene Expression in *Chlamydomonas reinhardtii*

In the present study, 27 independent cDNA libraries were constructed for individual samples of *C. reinhardtii* using a PE150 sequencing analysis. The number of reads mapped to each gene was determined using the featureCounts v1.5.0 p3. Subsequently, the length of the gene and read count mapped to this gene were used to calculate the fragment per kilobase of exon model per million mapped reads (FPKM) of each gene. Differential expression analysis of salt-treated and control samples at different time points was performed using the DESeq2 R package (Love et al., 2014). The false discovery rate was controlled by adjusting the *P*-value by Benjamini and Hochberg’s approach. Differentially expressed genes (DEGs) were defined using thresholds of *P*-value < 0.05 and | log2(foldchange)| > 0 and 1. The union of the DEGs was analyzed by the heatmap package of R for gene cluster construction, using the Euclidean distance clustering algorithm. The normalized expression values of DEGs were calculated by dividing their expression level at different time points with their maximum observed FPKM.

### Weighted Gene Co-expression Network Analysis Co-expression Network Construction

The FPKM values of genes were transformed and normalized to construct the weighted gene co-expression network analysis (WGCNA) co-expression network. The genes with a low variance of expression values among samples were omitted. A co-expression network for the filtered genes was constructed using the WGCNA package in R (Version 3.3.2) (Zhang and Horvath, 2005). After sample clustering, the soft threshold of module analysis was determined using the scale independence and mean connectivity analysis of modules with different power values. The power value ranged from 1 to 20, and then the values of scale independence and mean connectivity were calculated accordingly. The power value was calculated when the scale independence value was 0.9. To classify the similar gene expression profiles into different gene modules, the average distance with a minimum size threshold of 30 and the merge cut height of 0.25 were used to construct a hierarchical clustering dendrogram of the TOM matrix. Moreover, a cluster dendrogram among modules and an eigengene adjacency heatmap between modules were generated. Cytoscope 2.8.2 Shannon et al. (2003) was used to visualize the co-expression networks. Significant co-expression modules related to the salt stress response (as a trait) were identified using the information time-course NaCl treatment of 27 samples. The correlation between modules and traits was used to calculate the module–trait relationships, and the modules significantly associated with individual traits (*P*-value < 0.05) were identified.

### Gene Ontology Enrichment and Transcription Factor Screening

Based on the sequence similarity against the genome of *Arabidopsis thaliana* with an *E*-value cut-off of 1e^–5^, the genes of *C. reinhardtii* were mapped to *A. thaliana* gene IDs accordingly. The TF data of *A. thaliana* were downloaded from plantTFDB.^7^ The homologous of *A. thaliana* TFs *in C. reinhardtii* were screened as TFs in *C. reinhardtii* strain GY-D55. The Gene Ontology (GO) enrichment analysis for DEGs of different clusters and genes of co-expression modules was conducted by the topGO package of R.

### Quantitative Reverse Transcription PCR for Data Validation

Eight different genes were selected to verify the RNA-Seq result, including four upregulated genes (Pyrroline-5-carboxylate synthetase coding gene CHLREDRAFT_130812, Gal oxidase coding gene CHLREDRAFT_196816, Myb family TF CHLREDRAFT_205725, and WRKY family TF CHLREDRAFT_205718) and four downregulated genes (UDP-sulfoquinovose synthase coding gene CHLREDRAFT_27658, oxygen-evolving enhancer protein 2 of photosystem II coding gene CHLREDRAFT_33411, catalase coding gene CHLREDRAFT_190083, and L-ascorbate peroxidase coding gene CHLREDRAFT_192806). Gene sequences were extracted, and their primers were designed using Primer 5 software (Lalitha, 2000). RNA extraction and quality tests were performed as described above, and quantitative reverse transcription PCR (qRT-PCR) was performed according to the method described by Wang et al. (2018). Using the homolog of the GTP binding elongation factor Tu family protein EF1ALPHA (CHLREDRAFT_132905) as a reference internal gene. qRT-PCR was performed using SYBR Green qRT-PCR Master Mix (DBI, Ludwigshafen, Germany) in ABI7500 Real-Time PCR System (ABI, Waltham, MA, United States). Three replicates were performed, and the amplicons were used for melting curve analysis to evaluate the amplification specificity. Relative gene expression was quantified using the 2^–ΔΔCt^ method. The statistical differences between samples were analyzed by one-way ANOVA in R. The correlation coefficient of gene expression between qRT-PCR analysis and RNA-Seq data was analyzed in R.

## Results

### Effect of Salt on *Chlamydomonas reinhardtii* Strain GY-D55 Cell Growth

**FIGURE 1:**
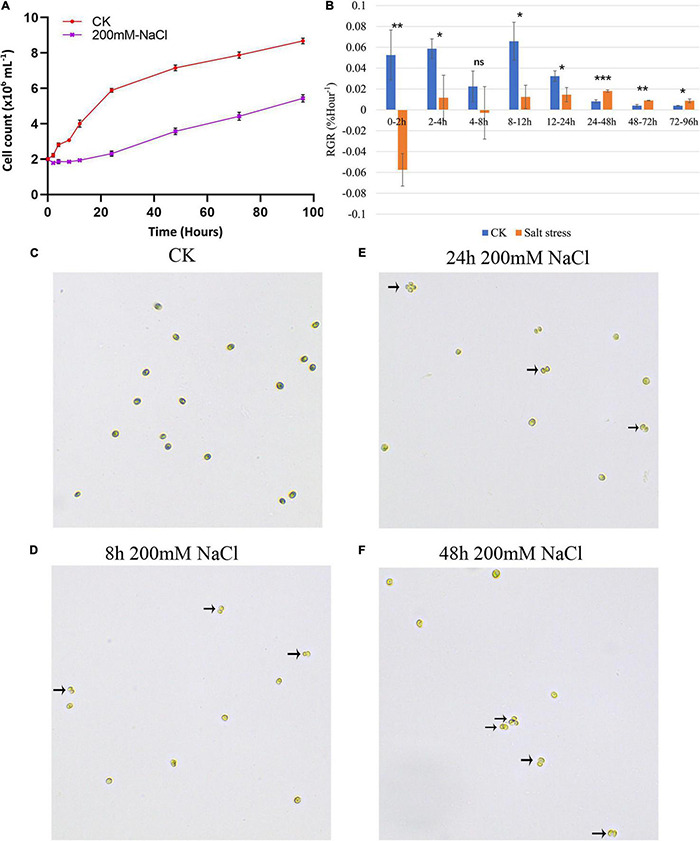
**(A)** Growth curves of GY-D55 strain during 0 (CK) and 200 mM sodium chloride (NaCl) stress. Growth curves are based on cell count. The scale bar corresponds to 10 μm. For all experiments *n* = 3. Error bars show SEM. The *t*-test results of growth rates in salt-treated samples and control samples at eight time-points were significant (*P*-value < 0.05). The morphology of *Chlamydomonas reinhardtii* GY-D55 cells: **(B)** changes in relative growth rates (RGR) of the NaCl treated and control groups. Means and SE are shown, *n* = 3. The statistical differences between salt stress and CK samples RGR were analyzed by one-way ANOVA in R. ***P < 0.001, **P < 0.01, *P < 0.05, ns means not significant. **(C)** Without addition of NaCl (CK), **(D)** 8 h under 200 mM NaCl treatment, **(E)** 24 h under NaCl treatment, **(F)** 48 h under NaCl treatment. The palmelloids were marked by a black arrow.

Growth analyses based on cell density showed that the growth rate of *C. reinhardtii* strain GY-D55 was detrimentally affected in the presence of 200 mM NaCl (Figure 1A and Supplementary Table 1). Moreover, the 44% reduction of growth rate was observed under the salt conditions at 0–96 h. Figure 1B shows that the RGR of the NaCl-treated and control groups in 0–2 h was significantly different (*P* < 0.01). In the NaCl-treated group, the RGR was negative during this period, indicating that the growth of GY-D55 cells was significantly inhibited in the early stage of salt stress. During 8–12 h, although the RGR of algae under salt stress was still significantly lower (*P* < 0.05) compared with the control group, GY-D55 began to gradually increase the number of cells during this period. After 48 h, the RGR of GY-D55 cells under salt stress was higher compared with the control group, although both values were very small. Besides, 200 mM NaCl exposure GY-D55 cells showed the time-dependent formation of palmelloids (Figures 1C–F). Two cell clusters were observed in the 4–8 h salt-treated samples (Figure 1D), four-celled clusters (akin to palmelloids) were found in cells under the 24–48 h NaCl treatment (Figures 1E,F), chlorophyll bleaching was found at the 2–12 h salt stress treated cells.

### Transcriptome Profiling and Novel Transcripts Discovery

The total mRNA from the cells of the three control and 24 salt stress treated samples of *C. reinhardtii* strain GY-D55 were sequenced using the Illumina system. The pairend reads obtained from 27 samples of *C. reinhardtii* were shown in Supplementary Table 2. In total, we obtained more than 100 million raw reads for each *C. reinhardtii* sample with at least 60 million reads for each condition and time point. More than 89% of the HQ reads from the individual sample (control and salinity) of *C. reinhardtii* strain GY-D55 could be mapped on the *C. reinhardtii* genome (Supplementary Table 2). The assembly of mapped reads resulted in the identification of a total of 19,267 transcripts in the *C. reinhardtii* strain GY-D55. Th identification of novel genes/transcript isoforms has emerged as one of the major advantages of RNA-sequencing analysis. We identified a total of 4,583 novel transcript isoforms in *C. reinhardtii* strain GY-D55.

### Time-Course Transcriptome of *Chlamydomonas reinhardtii* Strain GY-D55 in Response to Salt Stress

**FIGURE 2:**
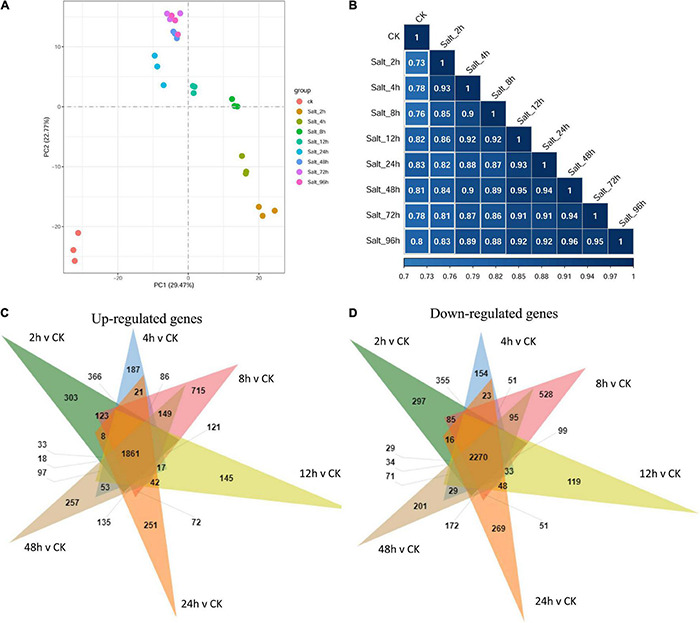
**(A)** The principal component analysis (PCA) plot and **(B)** gene expression correlation analysis using whole gene expression profiles of GY-D55 cells in different salt stress treated time points. The PCA shows the global gene expression of control samples was most distinct from the salt stress treated time points; gene expression of 2–24 h NaCl treated samples were scattered; samples of salt stress treated 48, 72, and 96 h clustered together. The correlation analysis indicated that gene expression of control samples was lowly correlated with the salt-treated samples and samples of late time points were highly correlated with each other. PCA, principal component analysis. **(C)** The Venn analysis result of upregulated genes for each time point compared with control samples. **(D)** The Venn analysis result of downregulated genes for each time point compared with control samples.

To elucidate the time-course of the transition in the gene expression of the *C. reinhardtii* strain GY-D55 under saline condition, we tracked changes in mRNA abundance by RNA-seq at control and time points (2, 4, 8, 12, 24, 48, 72, and 96 h) after alga cells were treated by 200 mM NaCl. Principal component analysis (PCA) indicated an apparent difference in gene expression between samples under control and different time-point salt stress. Clustering analysis using whole gene expression profiles showed an apparent time-dependent similarity in the profiles of the control and NaCl treated samples (Figure 2A): (1) the global gene expression of control samples was most distinct from the salt stress treated time points; (2) gene expression of 2–24 h NaCl treated samples were scattered; (3) samples of salt stress treated 48, 72, and 96 h clustered together, indicating these stages are closer at gene expression. This result is consistent with the gene expression correlation analysis (Figure 2B) that gene expression of control samples was lowly correlated with the salt-treated samples (*R*^2^ = 0.733–0.832) and samples of late time points were highly correlated with each other (*R*^2^ = 0.945–0.963).

### Detection of Differentially Expressed Genes

The relative expressions of genes in *C. reinhardtii* strain GY-D55 under control or salt stress treatments at eight time points were evaluated based on the FPKM values. Supplementary Table 3 shows the numbers of DEGs in the control samples and NaCl treated samples at eight time points. A total of 9,184 (up = 4,412/down = 4,772), 9,872 (4,887/4,985), 10,901 (5,555/5,346), 9,570 (4,702/4,868), 8,167 (3,805/4,362), 9,877 (4,880/4,997), 8,479 (4,055/4,424), and 9,766 (4,846/4,920) DEGs were identified in NaCl treated samples at eight time points compared with the control group (Supplementary Table 3). When the transcripts were compared at each time point under the saline condition, the number of DEGs between time points was the highest for 8 vs 48 h 9,776 (4,845/4,931) and 8 vs 96 h 9,244 (4,575/4,669), and the lowest for 72 vs 96 h 865 (371/494) and 48/h vs 72/h 2,760 (1,596/1,164), and there were little DEGs across NaCl treated samples among the time points of 48, 72, and 96 h (Supplementary Table 3). Overlapping studies found that there were 1,861/2,270 common upregulated/downregulated genes for each time point compared with control samples, and the overlapping details were shown in Figures 2C,D. Samples treated with salt for 2, 8, and 24 h had a relatively large number of characteristic upregulated/downregulated genes.

### Differentially Expressed Genes at Different Time Points and Function Enrichments

**FIGURE 3:**
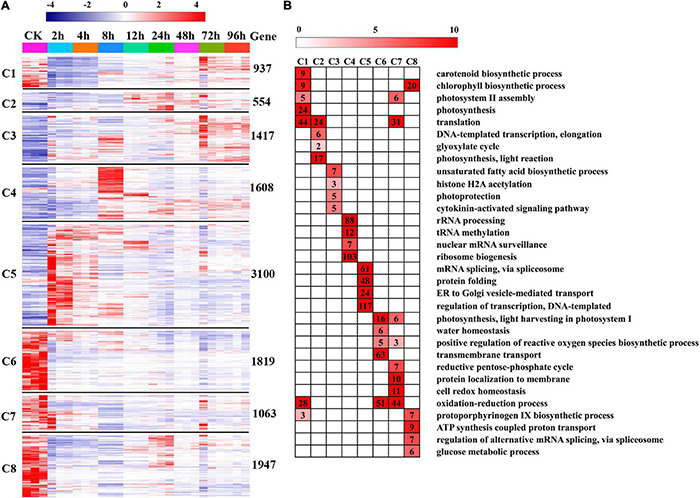
Gene expression pattern and functional transition over the time course. **(A)** Expression patterns of 12,445 differentially expressed genes (DEGs) genes in eight DEGs clusters. The number of genes in each cluster is shown on the right. **(B)** Gene Ontology (GO) enriched in eight DEGs clusters. Top four/five significant categories (*P*-value < 0.05) are displayed.

**FIGURE 4:**
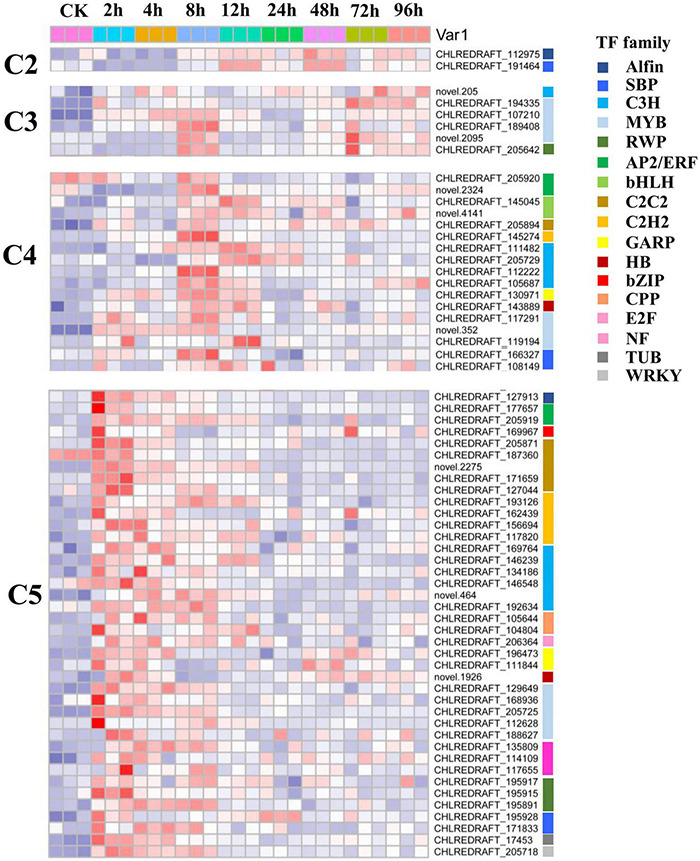
Expression pattern of transcription factors (TFs) including in DEGs cluster C2, C3, C4, and C5 in eight time points samples.

To further provide insights into the functional transitions along with salt stress response in *C. reinhardtii* strain GY-D55, we clustered the 12,445 DEGs into eight clusters using the Euclidean distance clustering algorithm (Figure 3A and Supplementary Table 4). The GO annotation was performed to assign genes to functional categories for each cluster (Figure 3B). Genes belonging to cluster 5 (C5) were mainly expressed at 2–4 h under the salt stress, and genes in clusters 6, 7, and 8 (C6, C7, and C8) were synchronously downregulated from 2 to 96 h under the saline stress. The early stage (2–4 h) was best represented by 3,100 expressed genes in C5 (Figures 3A,B). This cluster contained a set of genes related to “mRNA splicing, *via* spliceosome,” “protein folding,” “ER to Golgi vesicle-mediated transport,” and “regulation of transcription, DNA-templated” (Figure 3). Genes included in cluster 1 (C1) were downregulated at 2–8 h and participated in “carotenoid biosynthetic process,” “chlorophyll biosynthetic process,” and “photosystem II assembly” (Figure 3B and Supplementary Table 5). The genes in C6, C7, and C8 (1,819, 1,063, and 1,947 genes) were highly expressed in the control samples and represented by genes related to “photosynthesis, light harvesting in photosystem I,” “oxidation-reduction process,” and “ATP synthesis coupled proton transport” (Figure 3B and Supplementary Table 5). Inferred from the TFs data in *A. thaliana*, we identified a total of 113 TFs differentially expressed in salt stress responding in GY-D55 (Supplementary Table 6). Totals of 39.8% (45 of 113) and 19.4% (22 of 113) TFs were significantly enriched in DEGs clusters C5 and C4 (Figure 4).

### Weighted Gene Co-expression Network Analysis Co-expression Network Construction

A total of 18,417 genes were included in WGCNA. By sample clustering, no outliers were observed in 27 samples, thus all samples were included in the analysis. Then the soft threshold was determined by scale independence and mean connectivity analysis of modules with different power values ranging from 1 to 20. In our study, power = 7 was set to guarantee high scale independence (near 0.8) and low mean connectivity (near 0) (Supplementary Figure 1A). The mergeCutHeight was set as 0.25, and a total of 44 modules were generated and displayed with different colors (Supplementary Figure 1B). All analyzed genes were included in the 44 modules (Supplementary Table 7), 4,041 and 2,299 genes were assigned to the turquoise and blue module, red and magenta module included 864 and 515 genes, 314 genes were grouped to tan module.

### Module–Trait Relationship Calculation

The salt stress treated time points were collected as salt stress responding trait, and module–trait relationships were calculated according to the positive correlation between 44 modules and traits. The module trait relationship is shown in Supplementary Figure 2 and Table 1. There were five modules found positively associated with salt stress responding (Table 1). Module blue (cor = 0.592, 0.391, 0.435) was discovered associated with 2 h NaCl treated samples; the module brown (cor = 0.514, 0.509, 0.532) and magenta (cor = 0.384, 0.417, 0.451) were related with 8 h; module tan (cor = 0.429, 0.471, 0.505) were discovered associated with 12 h salt-treated samples; module darkturquoise (cor = 0.393, 0.396, 0.354) was related with 24h. Besides, module red was found mildly associated with 2-8 h salt-treated samples (Supplementary Figure 2).

**TABLE 1:** Module–trait relation and gene function enrichment information of genes in salt stress responding time points related modules.

Time	Module	Module–trait relation			GO ID	GO term	*P*-value
2 h	Blue	0.592 (0.001)	0.391 (0.04)	0.435 (0.02)	GO:0006468	Protein phosphorylation	6.50E−-1
					GO:0007165	Signal transduction	8.70E−06
					GO:0032784	Regulation of DNA-templated transcription, elongation	2.69E−03
8 h	Brown	0.514 (0.006)	0.509 (0.007)	0.532 (0.004)	GO:0006364	rRNA processing	6.40E−14
					GO:0030488	tRNA methylation	2.40E−06
					GO:0006351	Transcription, DNA-templated	3.10E−04
8 h	Magenta	0.384 (0.05)	0.417 (0.03)	0.451 (0.02)	GO:0032958	Inositol phosphate biosynthetic process	1.30E−03
					GO:0006813	Potassium ion transport	1.35E−02
					GO:0009734	Auxin-activated signaling pathway	1.71E−02
12 h	Tan	0.429 (0.03)	0.471 (0.01)	0.505 (0.007)	GO:0042256	Mature ribosome assembly	1.20E−04
					GO:0019761	Glucosinolate biosynthetic process	5.17E−03
					GO:0009098	Leucine biosynthetic process	7.63E−03
24 h	Darkturquoise	0.393 (0.04)	0.396 (0.04)	0.354 (0.07*)	GO:0009142	Nucleoside triphosphate biosynthetic process	1.20E−04
					GO:0006163	Purine nucleotide metabolic process	1.55E−03
					GO:0019722	Calcium-mediated signaling	4.18E−03

GO, Gene Ontology. *Represent the P-value > 0.05.

### Function Annotation of the Trait Related Modules

The results of the GO analysis of the filtered salt stress responding related modules were shown in Table 1 and Supplementary Table 8. For salt stress 2–4 h related module blue, we identified 47 GO terms, including “protein phosphorylation,” “signal transduction,” and “regulation of DNA-templated transcription, elongation.” A total of 45 terms were annotated in 8 h associated module magenta, including “inositol phosphate biosynthetic process,” “potassium ion transport,” and “auxin activated signaling pathway.” For 12 h related module tan, we identified 43 GO terms, including “mature ribosome assembly,” “glucosinolate biosynthetic process,” and “leucine biosynthetic process.” A total of 41 terms were annotated in 48–96 h associated module yellow, including “cellular response to red light,” “photoprotection,” and “chlorophyll biosynthetic process.” Besides, for salt stress 2-8 h negatively related module red, “reductive pentose phosphate cycle”, “chlorophyll biosynthetic process”, “oxidation-reduction process”, and “photosystem II assembly” were enriched (Supplementary Table 8).

### Transcription Factors Regulation in Weighted Gene Co-expression Network Analysis Co-expression Module Blue

A total of 1,192 DEGs cluster C5 genes were included in WGCNA co-expression module blue, including 17 TFs. Top 1% co-expression links of module blue were extracted for reducing complexity. The five C5 TFs included in the top 1% co-expression links in module blue are Alfin-CHLREDRAFT_127913, C2C2-CHLREDRAFT_205871, C2C2-CHLREDRAFT_171659, MYB-CHLREDRAFT_168936, and RWP-CHLREDRAFT_195915. Alfin-CHLREDRAFT_127913, C2C2-CHLREDRAFT_205871, and MYB-CHLREDRAFT_168936 were predicted to interact with 173, 72, and 6 genes, respectively, some of their potential target genes are overlapped.

### Real-Time Quantitative PCR Validation

To verify the RNA-seq results, an alternative strategy was selected for the upregulated genes. In total, eight genes were selected for validation by qRT-PCR using the same RNA samples that were used for RNA-sequencing (Supplementary Figure 3). The primers’ sequences were shown in Supplementary Table 9. In most cases, the gene expression trends were similar between these two methods, Pearson’s correlation of gene expression between qRT-PCR analysis and RNA-Seq data is cor = 0.765, *P*-_value < 3.464e^–07^; the result is shown in Supplementary Figure 3.

## Discussion

Existing research on *C. reinhardtii* shows that at the morphological level, salt stress of 150 or 200 mM NaCl results in flagellar resorption, decreased cell size, impaired growth, and palmelloid morphology (Neelam and Subramanyam, 2013; Khona et al., 2016). In the study published by Khona et al. (2016), the immediate death of *C. reinhardtii* cells was observed with 350 and 500 mM NaCl. We have tested the 300 M NaCl treatment on GY-D55 cells, a large number of cells death was observed with this concentration, which suggested the NaCl tolerance limits of green algae *C. reinhardtii* strain GY-D55. In the present study, the growth rate of *C. reinhardtii* strain GY-D55 was significantly impaired in the presence of 200 mM NaCl (Figure 1A), especially at early time points (2–12 h). Moreover, we characterized the formation of palmelloids in GY-D55 cells under salt stress. In land plants, cell-to-cell adhesion is regulated *via* a middle lamella mainly consisting of pectic polysaccharides. As a Gal oxidase, *RUBY* enhances pectin cohesion within the middle lamella of seed coat mucilage in *A. thaliana* (Sola et al., 2019). UDP-glucose pyrophosphorylase UGP1/2 is an enzyme playing a critical role in the metabolism of UDP-glucose, a precursor for the synthesis of carbohydrate cell wall components, including cellulose and callose (Klopffleisch et al., 2011). Besides, UDP-glucosyltransferase UGT-80 involves in the carbohydrate metabolic process. The upregulation of *RUBY* (CHLREDRAFT_196816) at 2–48 h, *UGT-80* (CHLREDRAFT_154976) at 2–12 h, and *UGP2* (CHLREDRAFT_157049) at 2–4 h in GY-D55 cells suggested the function of these genes in cell wall carbohydrates of palmelloid formation during the early stage of salt stress in microalgae.

Genes in DEG clusters C1, C6, and C8 were significantly downregulated at 2–4 h (Figure 3A), and the functional enrichment analyses of these genes suggested that short-term salt stress impaired oxidation-reduction, protein modification, water homeostasis, and chlorophyll biosynthesis of GY-D55 cells, which was consistent with the previous study. In the published studies, the cell growth and photosynthetic activity in several strains of *C. reinhardtii* recover over 24 h. In the present study, clusters C5, C4, C2, and C3 contained genes mainly expressed at 2–4, 8, 12, and 24 h, respectively (Figure 3A). We predicted the dynamic responding mechanisms of these clusters to salt stress in GY-D55 cells. The algal cells promoted the transcriptional regulation and protein folding to deal with protein synthesis/folding and other impairments caused by saline stress at 2–4 h. In 8 h, GY-D55 cells mainly regulated the synthesis and regulation of rRNA and tRNA to deal with salt stress. At 12 h, GY-D55 cells promoted transcription and translation and improved the expressions of photosynthetic genes to deal with the damage of photosynthesis. Light protection and cytokinin signaling pathways were activated in 24 h to promote the recovery of photosynthesis and regulate transcription.

*Chlamydomonas* response to abiotic stresses, including salt stress, has been investigated at both the proteomic and transcriptomic levels. Quantitative proteomic comparison of salt stress in *C. reinhardtii* strain CC-4325 and the snow alga *C. nivalis* reveals that salt stress enhances the accumulation of fatty acids in *C. nivalis* (Hounslow et al., 2021), *C. reinhardtii* does not have this response. The transcriptome analysis was used to investigate the molecular and genetic mechanisms of salt stress responses in *C. reinhardtii* strain GY-D55 after 24 h of acclimation to 200 mM NaCl (Wang et al., 2018). We compared several key genes’/proteins’ changes during salt stress in the above-mentioned studies and current work, and these genes/proteins are related to stress tolerance, photosynthesis, carbohydrate metabolism, and lipid metabolism (Table 2). In most cases, the gene expression trends were similar among the three studies. CC-4325 cells showed a higher abundance of HSP 70B after being treated with salt stress for 11 h, and the coding gene of HSP70B (CHLREDRAFT_126835) was upregulated at 8–96 h compared with control samples in GY-D55 cells. *HSP70B* has previously been shown to increase under salinity stress in salt-adapted *C. reinhardtii* cells and involve in the photosystem II damage (Sithtisarn et al., 2017) and repair process in *D. salina* (Goyal, 2007). These results suggest that it plays a similar role in salinity response in strain CC-4325 and GY-D55. Pyrroline-5-carboxylate synthetase (P5CS) catalyzes the first step in proline (Perrineau et al., 2014; Zhu, 2016) biosynthesis from glutamate, and we found that *P5CS2* (CHLREDRAFT_130812) was upregulated at 24 h in the analysis performed by Wang et al. (2018) and this work, suggesting that *P5CS2* regulated proline synthesis in modulating the salt stress tolerance in GY-D55 cells. In both transcriptomic analyses, the coding gene of glutathione S transferase (CHLREDRAFT_195543) was upregulated under the saline stress in GY-D55 cells, while many genes of the ROS-scavenging system were downregulated.

**TABLE 2:** Comparisons of gene/protein changes during salt stress in published studies and this study.

Gene ID	Gene name	Strain CC-4325 (Hounslow et al., 2021)		Strain GY-D55 (Wang et al., 2018)	Strain GY-D55				
		Salt_11 h vs 0 h	Salt_18 h vs CK_18 h	24 h vs CK	Salt_8 h vs CK(L_2_fc)	Salt_12 h vs CK(L_2_fc)	Salt_24 h vs CK(L_2_fc)	Salt_72 h vs CK(L_2_fc)	Salt_96 h vs CK(L_2_fc)
**Stress proteins**									
CHLREDRAFT_ 126835	Heat shock protein 70B	Up	–	Up	1.304	1.313	–	1.139	–
CHLREDRAFT_ 139760	BAX inhibitor 1	–	–	Up	–	1.343	–	1.052	1.096
CHLREDRAFT_ 130812	Pyrroline-5-carboxylate synthetase	na	na	Up	–	–	–	–	–
CHLREDRAFT_ 195543	Glutathione S transferase	na	na	Up	2.399	1.526	–	1.092	1.529
**Photosynthesis**									
CHLREDRAFT_ 187025	Light-harvesting chlorophyll-*a*/*b* protein of photosystem I	Down	Down	Down	−6.280	−3.426	−2.436	−2.514	−2.305
CHLREDRAFT_ 136294	Light-harvesting protein of photosystem I	Down	–	Down	−5.311	−2.723	−2.038	−2.008	−1.739
CHLREDRAFT_ 148921	Low-CO_2_-inducible protein	Down	–	–	−1.252	–	–	–	–
CHLREDRAFT_ 189430	Low-CO_2_-inducible chloroplast envelope protein	–	Up	Down	–	–	−1.669	1.355	–
CHLREDRAFT_ 153656	Oxygen evolving enhancer protein 3	–	Down	Down	−3.779	−2.105	−1.311	−1.494	−1.351
CHLREDRAFT_ 130316	Oxygen-evolving enhancer protein 1 of photosystem II	–	Down	Down	−3.598	−1.757	−1.168	−1.506	−1.381
CHLREDRAFT_ 33411	Oxygen-evolving enhancer protein 2 of photosystem II	–	Down	Down	−3.411	−1.543	–	–	–
CHLREDRAFT_ 153656	Oxygen-evolving enhancer protein 3, chloroplastic	–	Down	Down	−3.779	−2.105	−1.311	−1.494	−1.351
CHLREDRAFT_ 130914	Photosystem I reaction center subunit III	–	Down	Down	−3.658	−2.166	−1.796	−1.837	−1.836
CHLREDRAFT_ 112806	Photosystem II stability/assembly factor	–	Down	–	−1.409	–	–	–	–
CHLREDRAFT_ 82986	Ribulose bisphosphate carboxylase small chain	Down	–	–	−1.744	−1.392	–	–	–
**Carbohydrate**									
CHLREDRAFT_ 185081	UDP-glucose 6-dehydrogenase	Up	Up	–	–	–	–	1.472	1.378
CHLREDRAFT_ 196263	Pyruvate kinase	na	na	Up	1.384	1.296	–	1.068	1.203
CHLREDRAFT_ 196261	Pyruvate kinase	na	na	Up	1.252	–	–	–	–
CHLREDRAFT_ 118203	Pyruvate kinase	na	na	Up	–	–	1.022	–	–
**Lipid**									
CHLREDRAFT_ 94229	Glycerol-3-phosphate dehydrogenase	na	na	Up	1.053	–	–	–	–
CHLREDRAFT_ 146945	Glycerol-3-phosphate dehydrogenase	Up	Up	Up	3.869	4.947	3.636	3.934	3.890
CHLREDRAFT_ 146946	Glycerol-3-phosphate dehydrogenase (fragment)	Up	Up	Up	4.112	4.548	3.784	3.240	3.650
CHLREDRAFT_ 122970	Biotin carboxylase	–	Down	Down	−1.496	−1.192	−1.116	–	−1.045
CHLREDRAFT_ 27658	UDP-sulfoquinovose synthase	–	–	Down	−1.299	–	–	–	–

*“–” means the difference was not significant. L_2_fc, Log 2 fold change; Up, upregulated; Down, downregulated; na, not mentioned.*

Photosynthetic activity in *C. reinhardtii* is initially suppressed in the presence of 200 mM salt, while it recovers over 24 h (Crespo, 2012; Erickson et al., 2015). The CC-4325 and GY-D55 cells show decreased expressions of photosynthetic proteins/genes. Photosystem (PS)I reaction centers and light-harvesting protein were largely decreased, including photosystem I reaction center subunit III (CHLREDRAFT_130914) and light-harvesting chlorophyll-*a*/*b* protein of photosystem I (CHLREDRAFT_136294). Although the expressions of several PSI- and PSII-related genes slightly recovered after 24 h in GY-D55 cells (Supplementary Figure 4). PSI has a slower recovery than PSII once damage occurs. These impairments of PSI indicated longer-term damage to the photosynthetic apparatus in CC-4325 and GY-D55 cells. Oxygen evolving enhancer proteins of PSII remained unchanged at 11 h in CC-4325 cells, whilst it was decreased at 2 h in GY-D55 cells. This result indicated that PSII impairments occurred in GY-D55 cells early than CC-4325 cells. For alga, starch remobilization is important for starch storage to lipid storage and production of glycerol. UDP-glucose 6-dehydrogenase is an enzyme that catalyzes starch into glucose for use in glycolysis (Hounslow et al., 2021). Salt exposure caused a higher abundance of this protein over time in CC-4325 cells, while its coding gene (CHLREDRAFT_185081) was upregulated at a late stage (72–96 h) in this study and not altered in the study of Wang et al. (2018) in GY-D55 cells. Algae can divert cellular resources toward lipid accumulation under salt stress (Hounslow et al., 2021). UDP-sulfoquinovose synthase is involved in glycerolipid metabolism and thylakoid membrane sulfoquinovosyl diacylglycerol (SQDG) metabolism in algae. We found that the abundance of *SQDG* (CHLRE-DRAFT_27658) was reduced in GY-D55 cells at 2–8 h in our study, and it is downregulated at 24 h in the study of Wang et al. (2018), while it was not changed in CC-4325 cells. Although the depressed expression of *SQDG* provides a potential mechanism for a key difference between alga’s fatty acid responses, this strain did not display the lipid accumulation response to salt stress with the downregulation of fatty acid desaturase. This finding helped elucidate that what caused salt stress was a lipid trigger, but not *C. reinhardtii* strain GY-D55.

**FIGURE 5:**
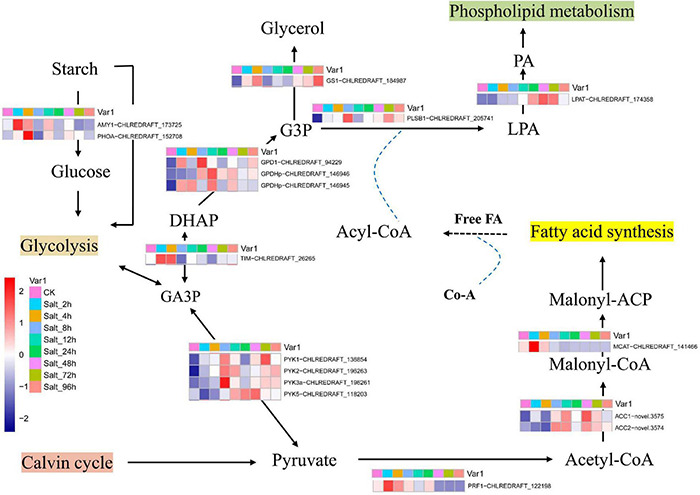
The schematic diagram of starch metabolism, glycerol production, and lipid synthesis. GA3P, glyceraldehyde3-phosphate; G3P, glycerol-3-phosphate; DHAP, dihydroxyacetone phosphate; LPA, lysophosphatidic acid; PA, phosphatidic acid. Mean FPKM values of the three samples in each time point were used as representative of genes’ expression value in eight salt-treated time points and control conditions.

Glycerol is an effective compatible solute that is produced by most salt-sensitive algal species under high saline stress (Goyal, 2007; Meijer et al., 2017; Hounslow et al., 2021). In the present study, the time course expressions of key genes were mapped into the schematic diagram of starch metabolism, glycerol production, and lipid synthesis (Figure 5). In the first step of glycolysis, starch is converted to glucose by alpha-amylase (AMY1, CHLREDRAFT_173725) (Shetty et al., 2019). The product of glycolysis is converted to glyceraldehyde-3-phosphate (GA3P) and then converted to dihydroxyacetone-phosphate (DHAP) by triosephosphate isomerase (Shetty et al., 2019) (TIM, CHLREDRAFT_26265). These two genes are mainly expressed at 2–4 h under salt stress in GY-D55 cells. Then, DHAP is converted to glycerol-3-phosphate (G3P) by glycerol-3-phosphate dehydrogenase (G3PDH). There are five isoforms of G3PDH enzyme in *C. reinhardtii* (Shetty et al., 2019). In the present study, two genes (GPD1, CHLREDRAFT_94229; GPDHp, CHLREDRAFT_146945) showed an over-expression pattern at 2–4 h under salt stress, and one gene (GPDHp, CHLREDRAFT_146946) was mainly expressed at 8–12 h. The homolog of glycerol-3-phosphatase (GS1, CHLREDRAFT_184987) catalyzes the formation of glycerol in higher plants (Singh et al., 2016; Shetty et al., 2019), and the homolog of this gene is upregulated at 4, 72, and 96 h in *C. reinhardtii*. The over-expression of key genes at 2–8 h under salt stress indicated that GY-D55 cells rapidly accumulated glycerol in the early stage of salt stress to cope with osmotic stress. There is an interplay between photosynthetic rate and the limited ability of the organism to utilize/export/store the photosynthetically fixed carbon. In the present study, photosynthesis was significantly inhibited before 24 h, which might be related to the accumulation of glycerol in the cells. Releasing large quantities of photosynthetic products leads to an increase in photosynthetic rates in plants. We speculated that GY-D55 cells leaked glycerol into the surrounding environment after 24 h to promote photosynthetic efficiency.

Transcription factor-encoding genes contribute to the diversity and evolution of plants. Identification of the TFs is essential in manipulating the regulatory network for abiotic stress in response to the target molecules (Lehti-Shiu et al., 2017). Inferred from the TF data in *A. thaliana*, we identified a total of 113 differentially expressed TFs in response to salt stress in GY-D55 (Supplementary Table 6). Moreover, 39.8 and 19.4% TFs were significantly enriched in DEG clusters C5 and C4, respectively, reflecting the important role of TFs in the early stage of salt stress in *C. reinhardtii* strain GY-D55 (Figure 4 and Supplementary Table 6). Several members from the bZIP, C3H, Dof, and MYB TF families were differentially expressed throughout the entire time course in salt and cold stresses. In the present study, eight/seven C3H TFs were upregulated at 2–4/8 h, respectively, indicating their functions in salt stress regulation. A total of five MYB TFs were clustered in C5, including a homolog of *MYB88* (CHLREDRAFT_205725). In *A. thaliana*, *MYB88* participates in the stomatal development in abiotic stress responses and the regulation of cell cycle genes (Xie et al., 2010). *Chlamydomonas* is a single-cell green alga, and there are no complex vegetative structures, such as leaves. However, TFs in plants have a higher retention rate after duplication compared with other genes (Lehti-Shiu et al., 2015; Thiriet-Rupert et al., 2016). Additionally, genes functionally related to stress responses tend to undergo a more intense duplication process (Riano-Pachon et al., 2008; Wu et al., 2015, 2021). Therefore, in higher plants and algae, the functions of genes in the same family or homologous group may be different, while they may participate in stress response conservatively.

**FIGURE 6:**
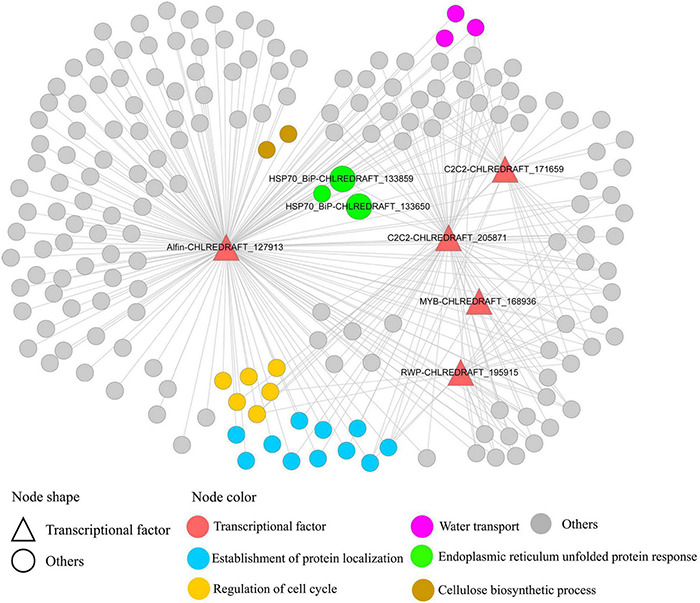
Five TFs regulating genes in co-expression module blue. This module was predicted to regulate endoplasmic reticulum (ER) stress at early time points. TFs (Alfin-CHLREDRAFT_127913, C2C2-CHLREDRAFT_205871, and C2C2-CHLREDRAFT_171659) regulating 11 genes of “establishment of protein localization” and three genes of “endoplasmic reticulum unfolded protein response,” including ER stress key gene *BiP* (CHLREDRAFT_133650 and CHLREDRAFT_133859).

Genes involved in related BPs tend to be co-expressed and clustered as functional modules (Ruprecht et al., 2017), which can help identify how the interplay between interconnected genes accomplishes specific biological functions. In the present study, “protein phosphorylation,” “signal transduction,” and “regulation of DNA-templated transcription, elongation” were top enriched BPs in the 2–4 h related module blue. Current evidence suggested that endoplasmic reticulum (ER) stress occurred at early time points (2–8 h) in GY-D55 cells. As shown in Figure 6, in module blue, Alfin-CHLREDRAFT_127913, C2C2-CHLREDRAFT_205871, and C2C2-CHLREDRAFT_171659 regulated 11 genes of “establishment of protein localization” and three genes of “endoplasmic reticulum unfolded protein response,” including ER stress key gene *BiP* (CHLREDRAFT_133650 and CHLREDRAFT_133859). As a member of the HSP70 family, *BiP* resides within the lumen of the ER, and it helps the folding and assembly of newly synthesized proteins as they are translocated into the ER and can also bind to misfolded, underglycosylated, or unassembled proteins (Crespo, 2012). Several members of Alfin-like TFs were upregulated in response to different abiotic stresses in *Brassica oleracea*. The role of Alfin-like TFs in enhancing salt stress and drought resistance is well-known when it is over-expressed in land plants roots. These results suggest the important role of the TFs of the Alfin and C2C2 families in salt stress in GY-D55 cells, especially in ER stress. In *C. reinhardtii*, most of the published studies have focused on the mechanisms of bZIP and MYB TFs in salt stress, while the TFs of other families are rarely reported (Ji et al., 2018). The present work provided a valuable foundation for TFs, benefiting the exploration of the regulatory network in algal abiotic stress.

## Conclusion

Here, we reported a comprehensive temporal investigation of transcriptomes using data at eight different time points, from early stage (2 h) to late stage (up to 96 h) in *C. reinhardtii* GY-D55 cells. A total of 12,445 genes were detected as DEGs. There were 1,861/2,270 common upregulated/downregulated genes for each time point compared with control samples. Samples treated with salt for 2, 8, and 24 h had a relatively large number of characteristic upregulated/downregulated genes. Short-time exposure to salt stress impaired oxidation reduction, protein synthesis and modification, and photosynthesis. The algal cells promoted transcriptional regulation and protein folding to deal with protein synthesis/modification impairments and rapidly accumulated glycerol in the early stage (2–4 h) to cope with osmotic stress. At 12 and 24 h, GY-D55 cells showed increased expressions of signaling and photosynthetic genes to deal with the damage of photosynthesis. The co-expression module blue was predicted to regulate ER stress at early time points. In addition, we identified a total of 113 TFs and predicted the potential roles of Alfin, C2C2, and MYB family TFs in algal salt stress responses.

## Data Availability Statement

The original contributions presented in the study are publicly available. This data can be found here: National Center for Biotechnology Information (NCBI) BioProject database under accession number PRJNA770825 and the Gene Expression Omnibus (GEO) database, GSE191218.

## Author Contributions

L-YZ and S-JF designed the experiments. L-YZ, Z-TX, and L-QC carried out the experiment and analyzed the data. L-YZ and Z-TX wrote the first draft of the manuscript. L-YZ, X-JZ, and S-JF supervised and completed the writing. All authors revised and approved the final manuscript.

## Conflict of Interest

The authors declare that the research was conducted in the absence of any commercial or financial relationships that could be construed as a potential conflict of interest.

## Publisher’s Note

All claims expressed in this article are solely those of the authors and do not necessarily represent those of their affiliated organizations, or those of the publisher, the editors and the reviewers. Any product that may be evaluated in this article, or claim that may be made by its manufacturer, is not guaranteed or endorsed by the publisher.

## Footnotes

1
http://www.leadingtec.cn/

2
https://submit.ncbi.nlm.nih.gov/subs/sra

3
https://www.ncbi.nlm.nih.gov/geo/

4
http://www.novogene.com/

5
ftp://ftp.ensemblgenomes.org/pub/release-39/plants/fasta/chlamydomonas_reinhardtii/dna/

6
ftp://ftp.ensemblgenomes.org/pub/release-39/plants/gtf/chlamydomonas_reinhardtii/

7
http://planttfdb.cbi.pku.edu.cn/

## Supplementary Material

Supplementary Figure 1 Construction of a weighted gene co-expression network. **(A)** Scale independence and **(B)** mean connectivity analysis. **(C)** Cluster dendrogram among modules.

Supplementary Figure 2 The salt stress treated time points expressed genes weighted gene co-expression network analysis (WGCNA) module–trait relationship. WGCNA, weighted gene co-expression network analysis.

Supplementary Figure 3 Validation of RNA-Sequencing (RNA-seq) data. Verification of the expression level of the selected eight differentially expressed genes (DEGs) from RNA-Seq data through RT-qPCR: **(A)** CHLREDRAFT_130812, **(B)** CHLREDRAFT_196816, **(C)** CHLREDRAFT_205725, **(D)** CHLREDRAFT_205718, **(E)** CHLREDRAFT_27658, **(F)** CHLREDRAFT_33411, **(G)** CHLREDRAFT_190083, **(H)** CHLREDRAFT_192806. Error bars indicate the standard error as mean + SD. The *x*-axis represents the relative expression level, and the *y*-axis represents NaCl treated time points. The statistical differences between salt treated and control samples were analyzed by one-way ANOVA. A total of 2, 12, 24, and 48 h were used as representative of eight time points in the RT-qPCR analysis. ^****^*P* < 0.0001, ^***^*P* < 0.001, ^**^*P* < 0.01, **P* < 0.05, ns means not significant. **(I)** Correlation coefficient of gene expression between qRT-PCR analysis and RNA-Seq data.

Supplementary Figure 4 The Kyoto Encyclopedia of Genes and Genomes (KEGG) pathways (ko00195) “photosynthesis” mapped with downregulated genes at different time points. The downregulated genes are marked by green color. The black solid line with a black arrow means molecular interaction or relation; the black dash line with a black arrow means indirect link or unknown reaction; the red dash line with a red arrow stands for the light quanta. The heatmaps of gene clusters were shown separately. A red color indicates that the gene is highly expressed under the corresponding treatment.

Supplementary Table 1 The *t*-test analysis result of the growth ratio between cells under control and saline condition.

Supplementary Table 2 Summary of mapping transcriptome reads to the reference sequence.

Supplementary Table 3 The differentially expressed gene number between the adjacent time point of salt stress responding in *Chlamydomonas reinhardtii* strain GY-D55.

Supplementary Table 4 The 12,445 differentially expressed genes in the eight clusters.

Supplementary Table 5 The Gene Ontology (GO) enrichment results of the eight differentially expressed gene clusters.

Supplementary Table 6 Summary of the differentially expressed transcription factors.

Supplementary Table 7 Gene number and edge number of 44 WGCNA modules.

Supplementary Table 8 The GO enrichment results of 44 WGCNA modules’ members.

Supplementary Table 9 Information of the quantitative real time PCR (qRT-PCR) primers.
