# Supplementary figures and images for: Comprehensive Time-Course Transcriptome and Co-expression Network Analyses Identify Salt Stress Responding Mechanisms in Chlamydomonas reinhardtii Strain GY-D55
Source: Front Plant Sci. 2022 Feb 24;13:828321. doi: 10.3389/fpls.2022.828321 (PMC8908243; doi:10.3389/fpls.2022.828321)

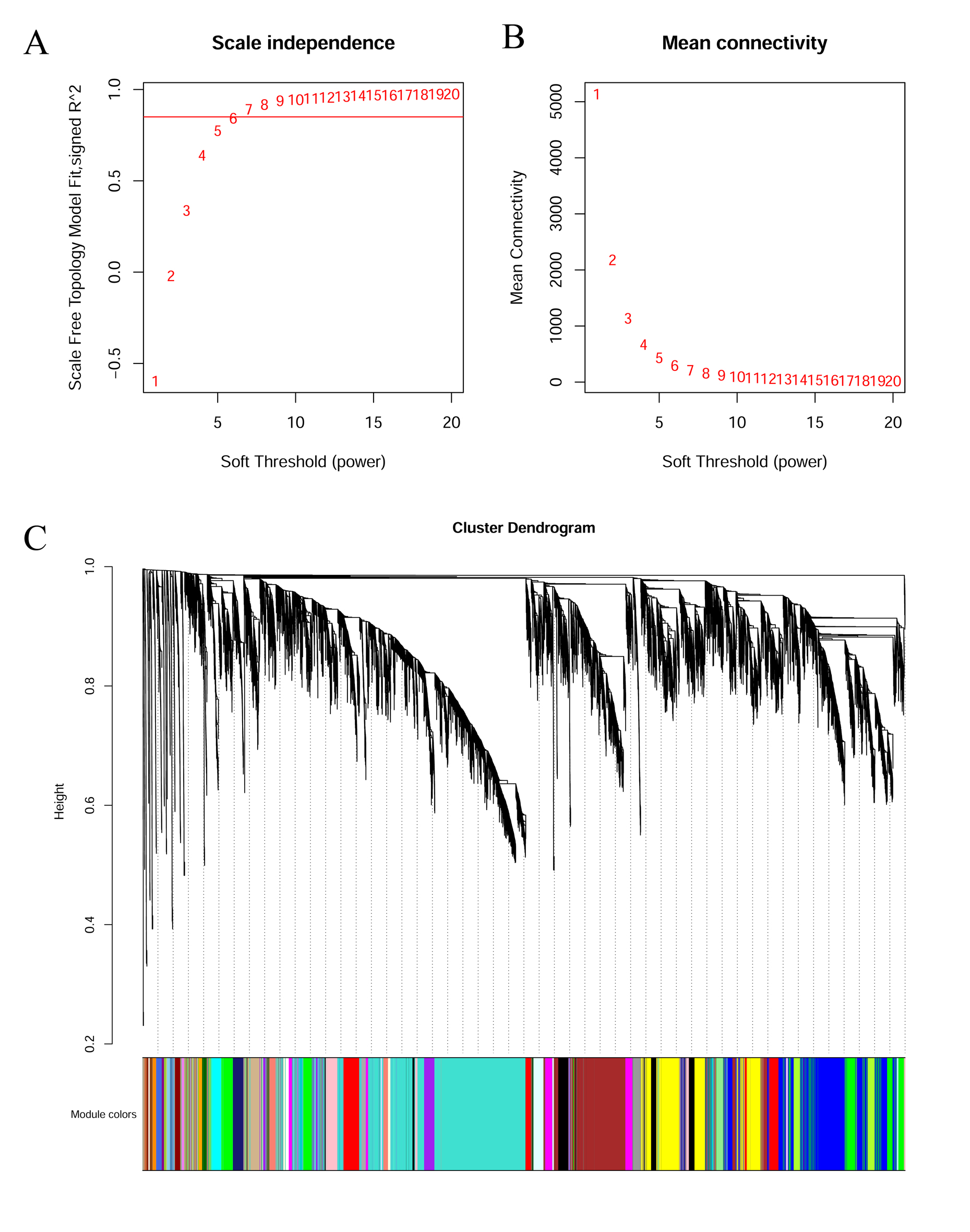

Supplement: Supplementary Figure 1 — Construction of a weighted gene co-expression network. (A) Scale independence and (B) mean connectivity analysis. (C) Cluster dendrogram among modules. [file Image_1.JPEG]

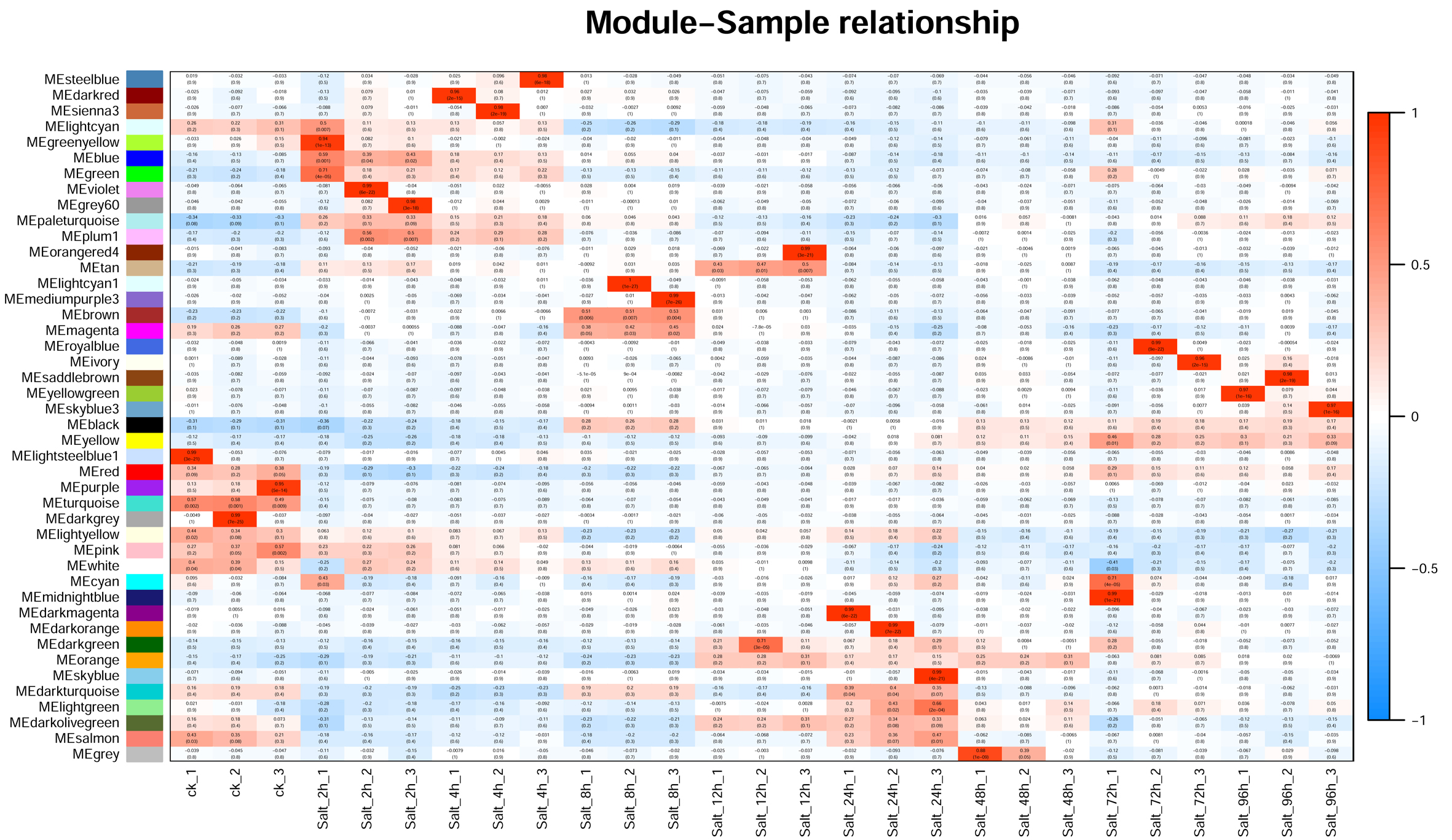

Supplement: Supplementary Figure 2 — The salt stress treated time points expressed genes weighted gene co-expression network analysis (WGCNA) module–trait relationship. WGCNA, weighted gene co-expression network analysis. [file Image_2.JPEG]

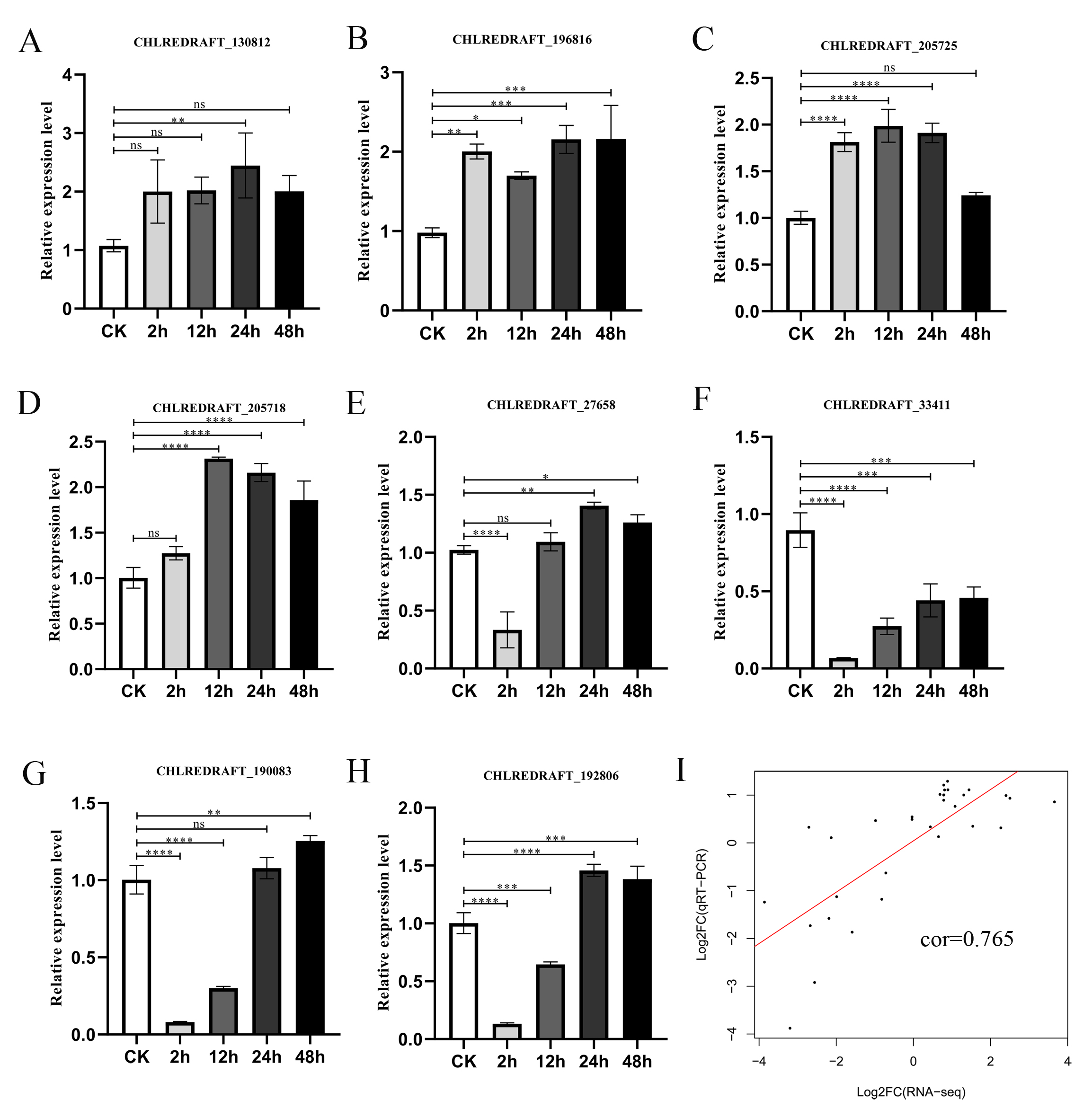

Supplement: Supplementary Figure 3 — Validation of RNA-Sequencing (RNA-seq) data. Verification of the expression level of the selected eight differentially expressed genes (DEGs) from RNA-Seq data through RT-qPCR: (A) CHLREDRAFT_130812, (B) CHLREDRAFT_196816, (C) CHLREDRAFT_205725, (D) CHLREDRAFT_205718, (E) CHLREDRAFT_27658, (F) CHLREDRAFT_33411, (G) CHLREDRAFT_190083, (H) CHLREDRAFT_192806. Error bars indicate the standard error as mean + SD. The x-axis represents the relative expression level, and the y-axis represents NaCl treated time points. The statistical differences between salt treated and control samples were analyzed by one-way ANOVA. A total of 2, 12, 24, and 48 h were used as representative of eight time points in the RT-qPCR analysis. ****P < 0.0001, ***P < 0.001, **P < 0.01, *P < 0.05, ns means not significant. (I) Correlation coefficient of gene expression between qRT-PCR analysis and RNA-Seq data. [file Image_3.JPEG]

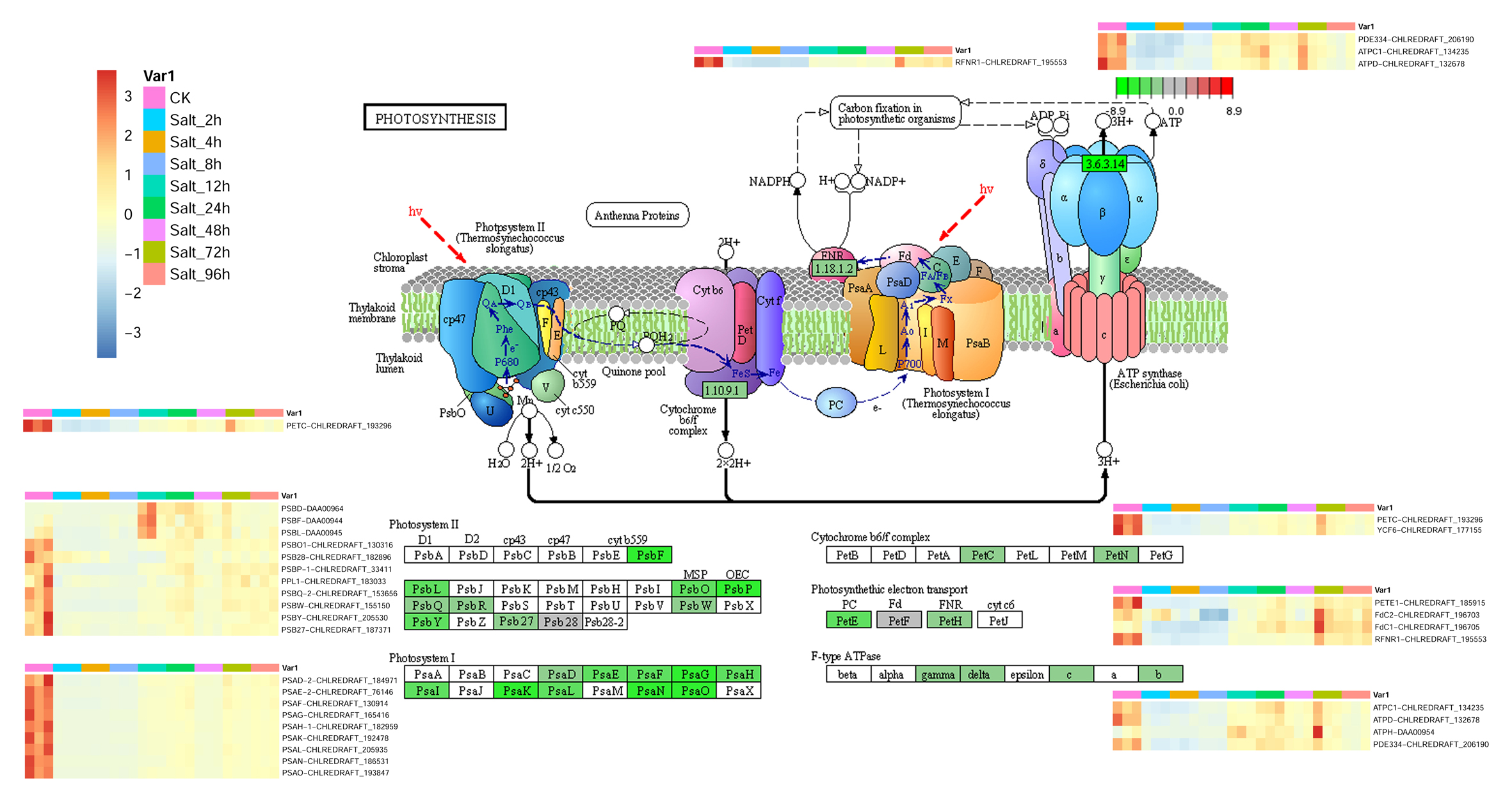

Supplement: Supplementary Figure 4 — The Kyoto Encyclopedia of Genes and Genomes (KEGG) pathways (ko00195) “photosynthesis” mapped with downregulated genes at different time points. The downregulated genes are marked by green color. The black solid line with a black arrow means molecular interaction or relation; the black dash line with a black arrow means indirect link or unknown reaction; the red dash line with a red arrow stands for the light quanta. The heatmaps of gene clusters were shown separately. A red color indicates that the gene is highly expressed under the corresponding treatment. [file Image_4.JPEG]
